# Three-Dimensional Mapping-Aided Global Navigation Satellite System in Global Navigation Satellite System-Accessible Indoor Areas

**DOI:** 10.3390/s26031058

**Published:** 2026-02-06

**Authors:** Hoi-Wah Ng, Hoi-Fung Ng, Li-Ta Hsu, John-Ross Rizzo

**Affiliations:** 1 Department of Aeronautical and Aviation Engineering, The Hong Kong Polytechnic University, 11 Yuk Choi Rd, Hung Hom, Hong Kong, China; hoi-wah.ng@connect.polyu.hk (H.-W.N.); lt.hsu@polyu.edu.hk (L.-T.H.); 2 Department of Physical Medicine and Rehabilitation, New York University Langone Health, New York, NY 10016, USA; johnrossrizzo@gmail.com; 3 Department of Neurology, New York University Langone Health, New York, NY 10016, USA; 4 Department of Ophthalmology, Grossman School of Medicine, New York University, New York, NY 10016, USA; 5 The Institute for Excellence in Health Equity, Grossman School of Medicine, New York University, New York, NY 10016, USA; 6 Department of Biomedical Engineering, Tandon School of Engineering, New York University, New York, NY 11201, USA; 7 Department of Mechanical and Aerospace Engineering, Tandon School of Engineering, New York University, New York, NY 11201, USA

**Keywords:** 3DMA GNSS, indoor positioning, seamless positioning, BIM, LiDAR

## Abstract

**Highlights:**

**What are the main findings?**

- The integration of Three-Dimensional Mapping-Aided (3DMA) Global Navigation Satellite System (GNSS) and Pedestrian Dead Reckoning (PDR) increases the accuracy and availability of positioning solutions in dense urban and indoor spaces.
- When comparing with general 3DMA, indoor 3DMA GNSS requires more accurate 3D models to increase the robustness of positioning solutions.

**What are the implications of the main finding?**

- Results of the study highlight the potential of the integrated approach for positioning in GNSS limited areas and during GNSS outages.
- Further studies should be conducted to reduce the overall error budget of positioning solutions in dense urban and indoor areas.

**Abstract:**

The Global Navigation Satellite System (GNSS) is commonly used for outdoor positioning. However, its effectiveness diminishes in urban canyons and indoor environments attributed to signal blockage. This study aims to explore the potential of GNSS signals penetrating indoor spaces through windows and to enhance indoor positioning with Three-Dimensional Mapping-Aided (3DMA) GNSS, a concept generally applied outdoors. The research employs a 3D model of a corridor with manually labeled window locations to predict satellite visibility within indoor areas. The study integrates Pedestrian Dead Reckoning (PDR) with an indoor Shadow-matching (I-SM) technique, utilizing an Extended Kalman Filter (EKF) to improve positioning accuracy. One of the findings indicates that the proposed method significantly enhances positioning performance and its availability, achieving a root mean square error (RMSE) that is 2 m better than using PDR alone or single epoch I-SM. The study concludes that integrating GNSS with I-SM technique and PDR can optimize an indoor positioning solution and highlights the potential for improved navigation solutions in complex urban environments.

## 1. Introduction

**Figure 1:**
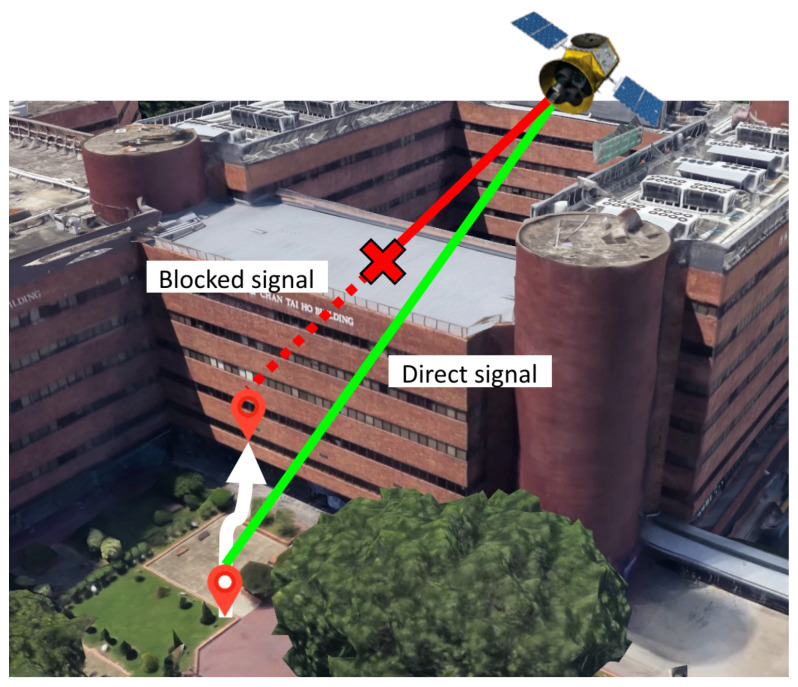
Direct (green) and blocked (red) GNSS signals in urban environments.

Positioning devices are usually installed with a Fused Localization System that contains multiple sensors to receive different signals for wireless positioning in outdoor and indoor environments. The Global Navigation Satellite System (GNSS) and the cellular network or mobile stations [1] are widely used for positioning outdoors, providing users with reliable location data across various applications. However, GNSS performance is often degraded in dense urban settings due to signal blockage and interference [2,3], limited satellite visibility, and poor satellite geometry [4]. Figure 1 illustrates the common problem of GNSS signal obstruction in urban areas. In indoor environments, GNSS is generally unable to offer valid localization results due to signal occlusion, attenuation, and the multipath effect [5]. Therefore, when moving into an indoor environment, the system tends to switch to other wireless positioning technologies available, such as Wi-Fi fingerprinting [6], Ultra-Wideband (UWB) [7], Bluetooth Low Energy (BLE) [8], and Pedestrian Dead Reckoning (PDR) [9]. They typically provide position information relative to known points in the indoor space within a local reference frame. For reliable and seamless positioning in both environments, the previous literature advocates the coupling of GNSS and Wi-Fi given the widespread availability [10]. Nevertheless, apart from offering relative positioning information, Wi-Fi positioning is less reliable because Wi-Fi is primarily designed for local area networking and Internet access, not precise location tracking. Wi-Fi access points (APs) might suffer from poor spatial geometry because owners typically install a minimal number of APs to reduce the installation and maintenance cost [11]. This may deteriorate the signal quality [12] and compromise their effectiveness for precise positioning. In contrast, GNSS delivers solutions in an absolute coordinate system in which all points are referenced to a fixed origin. All sets of coordinates in it are unique and globally fixed.

The need for an innovative solution arises when determining a user’s position where GNSS is traditionally unreliable or denied, specifically when the user is along indoor corridors with windows on one side or covered walkways, where part of the GNSS constellation is visible. Integrating GNSS and indoor localization technologies increases overall positioning accuracy by allowing their measurements to compensate for each other’s limitations. In indoor spaces, when GNSS signals cannot be received, indoor positioning techniques can still provide navigation prompting. Practically, it is essential to adopt absolute positioning in indoor navigation. In urban areas, it is common for users to transition between outdoor and indoor areas. It is more consistent to utilize the same frame of reference for localization in both settings in terms of navigation continuity and coordinate representation. Meanwhile, in the context of seamless positioning, it is more efficient to deploy absolute positioning and align with the global coordinate system, the same as that in the outdoor environment. Less coordinate transformation time is needed when moving between indoor and outdoor areas if both settings adopt an identical coordinate architecture. It enhances the positioning experience and performance. Economically, this integrated approach might reduce the capital allocated for the installation of navigational sensors. Three-Dimensional Mapping-Aided (3DMA) GNSS is a software-based method and does not require additional sensors during implementation. The user can obtain optimized coordinates once GNSS satellite signals are received and 3D models are available. It eliminates the need and expense for stakeholders to install indoor sensors for indoor positioning.

The concept of 3DMA GNSS was developed and implemented to improve the positioning accuracy in mainly outdoor areas in dense urban environments [13,14,15,16,17,18,19,20,21,22]. It integrates 3D city models and features of GNSS measurements to optimize the initial position guess.

The idea can be divided into Shadow-matching (SM) [13,14,15,16,17] and ranging-based 3DMA or likelihood-based ranging (LBR) [18,19,20]. The former compares the received carrier-to-noise ratio ($C/N_{0}$) with the predicted GNSS satellite visibility [15]. Commonly, building boundaries are extracted from a 3D city model to predict the visibility of GNSS satellites from the user’s perspective. The satellite is considered visible if the line-of-sight (LOS) signal is clear of building edges; otherwise, it is predicted to be non-line-of-sight (NLOS). In addition, the measured satellite visibility is determined by the received $C/N_{0}$ value. A satellite is classified as visible if its $C/N_{0}$ value is above a certain threshold; otherwise, it is categorized as invisible. The actual GNSS satellite visibility can also be resolved using probability theory and statistical models [16]. This information is used and combined when calculating the weighted scores to be assigned to every candidate. The scoring scheme in [17] further considers signal diffraction and reflection. The one with the highest score, which generally involves a reliable match in predicted and measured visibility, is chosen as the final position estimate.

Various ranging-based 3DMA methods have been implemented with GNSS pseudorange measurements [18,19,20]. Those methods detect and correct ranging errors caused by NLOS signals predicted by 3D models or exclude them during localization [18]. Some research exploits NLOS signals to generate hypotheses to be tested. In [19], reflection points are identified with the building boundaries to simulate reflected signals. Meanwhile, NLOS signal errors are modeled and corrected in [20]. GNSS satellite visibility is first determined using the 3D model of the city. After subtracting all pseudoranges from known errors, NLOS signals are mapped to LOS signals by statistically modeling different combinations of error distributions.

Based on the above 3DMA approaches, University College London (UCL) developed a hypothesis-domain integrated 3DMA GNSS algorithm [14]. It integrated both methods, SM and LBR, and determined the final position solution by the combined scores of candidates. Improvements are made to further enhance the accuracy and reliability of 3DMA GNSS positioning results. Grid filtering and particle filtering are applied to multi-epoch 3DMA GNSS positioning to optimize the results in urban environments [14]. Furthermore, outliers are detected and filtered to reduce the effects of temporary environmental changes when producing the map [21]. Recently, in [22], the authors proposed solutions to address existing limitations. For SM, upper and lower elevation constraints are introduced to address overhanging structures in cities. In addition, instead of ignoring undetected GNSS signals, they are deployed to improve satellite geometry. Moreover, the satellite visibility model is updated to adapt to diverse conditions, such as dense urban areas and open-sky areas, to avoid adjusting model parameters separately for different environments. For LBR, the work introduced an improved model to simulate NLOS path delay with $C/N_{0}$ of measurements. Meanwhile, a clustering-based method is deployed to determine the final position estimate. Issues of shifting and ambiguity arise when accumulating substantial low-scoring candidates and assigning the centroid of the search region as the final solution, respectively. These make the solution deviate from the correct position. The paper filtered low-score candidates and segmented various high-scoring regions when computing the solution. Weighted position solutions are calculated for each region consisting of high-scoring candidates. The one closest to the conventional GNSS-derived solution is selected as the final position estimate.

Wearable navigation systems have been developed for people with blindness and low vision (BLV) to navigate in urban areas [23]. Firstly, Visually Impaired Smart Service System for Spatial Intelligence and Navigation (VIS^4^ION) is a backpack wearable system for visually impaired persons to navigate in both outdoor and indoor environments. It consists of high-resolution cameras, microphones, and inertial measurement units (IMUs) connected to a portable computer for real-time analysis. Meanwhile, a multi-sensor integration approach is also proposed for persons with BLV. SM is integrated with LBR to generate positioning hypothesis candidates. Then, the 3DMA GNSS solution is integrated with velocity data estimated by Doppler measurements using factor graph optimization (FGO). It is concluded that this coupled algorithm provides a robust positioning performance in urban canyons.

This study potentially expands the applicable area of 3DMA GNSS from outdoor settings into GNSS-accessible indoor environments. Previous studies on 3DMA GNSS predominantly concentrated on outdoor environments. Urban users frequently enter indoor spaces that are partially visible to GNSS satellites. In such scenarios, it is hypothesized that 3DMA GNSS can still deliver reliable positioning results despite challenging settings. However, this aspect lacks systematic investigation. This paper presents a feasibility study on applying the 3DMA GNSS concept, specifically SM, to GNSS-accessible indoor environments. The core objective is to leverage the distinctive satellite visibility features within an indoor space, such as a corridor with windows on one side where signals can penetrate through them, to determine a user’s position. In such environments, the azimuth angle of a satellite can help determine the user’s longitudinal position along the corridor, while the elevation angle provides information about the lateral offset and height of their position. The research exploits the concept of Building Information Modeling (BIM) and investigates how a 3D model of a corridor, generated from a Light Detection and Ranging (LiDAR) point cloud scan, can be used to predict satellite visibility and match it with actual GNSS measurements to achieve localization.

The proposed positioning framework is based on a loose coupling of indoor Shadow-matching (I-SM) and PDR. The process begins by sampling numerous position hypothesis-candidates within a 3D floor plan. Each candidate is assigned a score depending on the similarity between the predicted satellite visibility from the 3D model and the observed $C/N_{0}$ measurements. Concurrently, PDR utilizes accelerometer readings to estimate the user’s displacement by detecting steps and calculating the stride length and heading. This trajectory derived from PDR provides a temporal constraint that is crucial for selecting the most probable position cluster over time, with an Extended Kalman Filter (EKF) [24] being used to integrate the information and update the final-state estimate. The framework focuses primarily on recovering the 2D positioning accuracy because GNSS is generally less accurate in measuring the vertical dimension than in the horizontal direction. The vertical displacement between steps is usually determined by other sensors.

The remainder of this paper is organized as follows: Section 2 introduces the proposed positioning framework in urban canyons; Section 3 presents and evaluates the results; Section 4 discusses the results; Section 5 concludes the work and suggests future directions.

## 2. Experimental Set-Up and Data Acquisition

This study aims to investigate the feasibility of the concept of 3DMA GNSS in indoor positioning, specifically focusing on dense urban and indoor environments identified as GNSS-limited areas with windows. The proposed positioning framework is based on the coupling of I-SM and PDR. It integrates the positions of GNSS satellites and their $C/N_{0}$ measurements with accelerometer readings and heading information within an EKF to produce a final-state estimate.

### 2.1. Study Area

**Figure 2:**
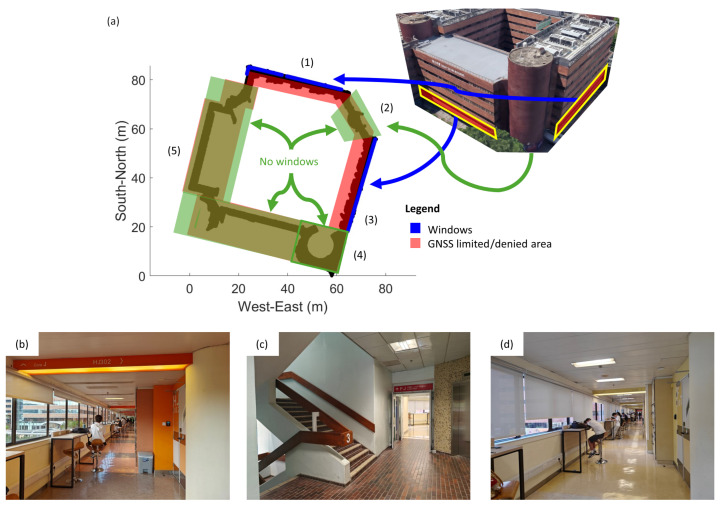
The study area: (**a**) Top-view of the study area. (**b**) Photo of (1). (**c**) Photo of (2). (**d**) Photo of (3).

**Figure 3:**
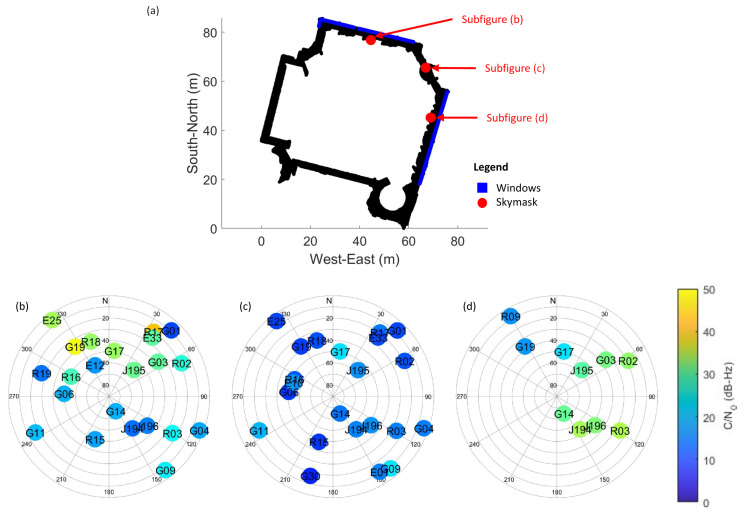
Skymasks generated along different parts of the corridor, colored with the $C/N_{0}$ values: (**a**) Location of skymasks. (**b**) The skymask at (1), where satellites in the north and northeast have higher $C/N_{0}$ values. (**c**) The skymask at (2). (**d**) The skymask at (3), when $C/N_{0}$ values of satellites in the east and southeast are higher.

The experiment was conducted along an angled corridor on the campus of The Hong Kong Polytechnic University (PolyU). Figure 2 and Figure 3 are maps and photos of the study area and skymasks generated in the parts of the corridor, respectively. Figure 2a outlines the setting of the study area. The black region represents the corridor of the building. The experiment was conducted along an angled corridor, which can be divided into five GNSS-limited or denied sections (Sections (1)–(5)). Windows are indicated as blue lines in the figure. There are windows in Sections (1) and (3) but not in Sections (2), (4), and (5). Figure 2b–d shows the interior view of Sections (1)–(3). Figure 3 illustrates the distribution of GNSS satellites and the quality of GNSS signals received at Sections (1)–(3) of the corridor during the first run of this study. In each skymask, concentric circles represent the elevation angles, ranging from $0^{\circ }$ to $90^{\circ }$, from the device to the GNSS satellites. Radial lines point out their azimuth angles between $0^{\circ }$ and $360^{\circ }$. Each colored circle denotes a GNSS satellite. It is labeled with the satellite system identifier (G: GPS, R: GLO, E: Galileo, and J: QZSS) and the PRN number of the satellite. The color of the circle corresponds to the $C/N_{0}$ value of the GNSS signal in dB-Hz, as indicated by the color bar on the right.

Signal conditions vary across different parts of the corridor. In Section (1), the windows are on the north side of the corridor. GNSS signals can penetrate through the windows, so in the epoch, the $C/N_{0}$ values of visible satellites on the north are relatively high. Section (2) is a transition area between Sections (1) and (3) and there are no windows along this section. All $C/N_{0}$ values are low since GNSS signals are obstructed by concrete. In Section (3), the windows are on the southeast side of the corridor. The $C/N_{0}$ values of satellites on that side are higher.

### 2.2. Three-Dimensional Model Construction

To facilitate the 3DMA GNSS concept, a detailed 3D model of the study area was constructed with point cloud scanning. This was performed using various LiDAR systems, specifically HDL 32E Velodyne, VLP16 Velodyne, and LSLiDAR C16. These sensors capture the physical geometry of the indoor environment as a high-density point cloud. The raw point cloud data were then post-processed with LIO-SAM [25], a system for LiDAR inertial odometry and mapping, to generate a coherent and accurate 3D representation of the environment. After that, the self-scanned point cloud was placed in the local coordinate system. The locations of the windows of the building were manually labeled within the 3D model using MATLAB 9.13.0 (R2022b) [26]. This was crucial because these distinct features, the primary pathway for the penetration of GNSS signals into the indoor space, help predict satellite visibility. Figure 3b–d involves examples of skymasks produced during the first run of the study. They contain satellites colored with their $C/N_{0}$ values.

### 2.3. GNSS Data Collection

For actual GNSS measurements to validate the system, the Xiaomi Mi 8 and Google Pixel 9 Pro XL smartphones were utilized to collect real-world satellite data. These setups allow the assessment of the actual reception and strength of the signal within the context of the prepared 3D model. These elements formed the basis for testing the proposed positioning framework, which incorporates hypothesis position candidates in a 3D space, followed by the clustering and integration with PDR.

### 2.4. Ground Truth Generation

Ground truth data are generated during the data collection process to evaluate the performance of the proposed positioning framework. The Share SLAM S20 SE was employed to capture the trajectory of the user during the experiment. It is a 3D LiDAR scanner with cameras, IMU, and laser scanners. The device can capture photos and emit laser pulses to scan the environment and record the user’s trajectory and movement under specific frequencies. In this experiment, the frequency is set to 1 Hz. The generated rosbag files and photos are then post-processed by Share UAV’s LiDAR Simultaneous Localization and Mapping (SLAM) and Visual SLAM algorithms to generate a trajectory file and point cloud data. However, they are in another local coordinate system. Therefore, coordinate transformation is performed to align the trajectory with the self-scanned 3D model in Section 2.2. The new point cloud data are translated and rotated about the Z-axis to acquire a rotation matrix in CloudCompare [27]. The rotation considers only the rotation about the Z-axis, as the framework focuses on the horizontal coordinates of points but not their elevation. The rotation matrix is multiplied by the translated trajectory data to ensure that it is in the same local coordinate system (Equation (1)). The transformed trajectory is then used as ground truth to evaluate the performance of the proposed framework.

(1) $${\left[\begin{array}{c} X\\ Y\\ Z \end{array}\right]}_{new}=\left[\begin{array}{c} \Delta X\\ \Delta Y\\ \Delta Z \end{array}\right]+\left[\begin{array}{ccc} \cos\theta & -\sin\theta & 0\\ \sin\theta & \cos\theta & 0\\ 0 & 0 & 1 \end{array}\right]{\left[\begin{array}{c} X\\ Y\\ Z \end{array}\right]}_{old}$$

where ΔX, ΔY, and ΔZ are translation parameters in the X, Y, Z directions, respectively, and θ is the rotation angle about the Z-axis (positive for a counterclockwise rotation).

The results demonstrate the potential of this integrated approach to provide a positioning solution during GNSS outages. In the optimal case, the integration with PDR achieves a 2D root mean square error (RMSE) of 5.52 m, approximately 2 m better than solely PDR and single epoch I-SM alone (7.44 m and 7.79 m, respectively), proving to be effective in selecting the correct position cluster, particularly when navigating turns or experiencing signal loss. However, the study also highlights significant challenges. Performance is sensitive to inaccuracies in the 3D model, for example, the presence of dynamic obstacles such as humans or signal-penetrable materials like curtains, which are not accounted for in a static map. Furthermore, errors in GNSS observations, including incorrect $C/N_{0}$ values, can lead to mismatches between predicted and actual satellite visibility. Finally, the accuracy of the PDR system, especially its heading estimation, is critical as incorrect heading can deteriorate the overall system performance. This work contributes a foundational study on adapting the SM technique for indoor use, establishing a complete positioning framework, and identifying key error sources for future development.

## 3. Positioning Framework

**Figure 4:**
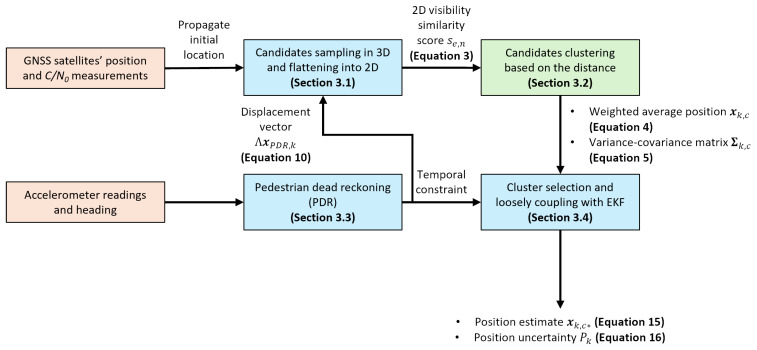
Flowchart of the proposed position framework.

The proposed positioning algorithm is designed to recover a user’s location in GNSS-denied or unreliable indoor areas by leveraging 3D building models. The framework is based on a loose coupling of I-SM and PDR, which provides essential temporal constraints for robust localization. The initial hypothesis sampling is performed in 3D to account for the height sensitivity. This framework concentrates on 2D horizontal positioning accuracy, following most GNSS applications and to leverage GNSS precision in 2D positioning. The overall process integrates GNSS satellite position and $C/N_{0}$ measurements with accelerometer readings and heading information within an EKF to produce a final-state estimate. The workflow consists of four stages: (1) position hypotheses sampling, (2) candidate clustering, (3) PDR estimation, and (4) cluster selection with EKF integration. Figure 4 summarizes the process.

### 3.1. Positioning Hypothesis Candidate Sampling

**Figure 5:**
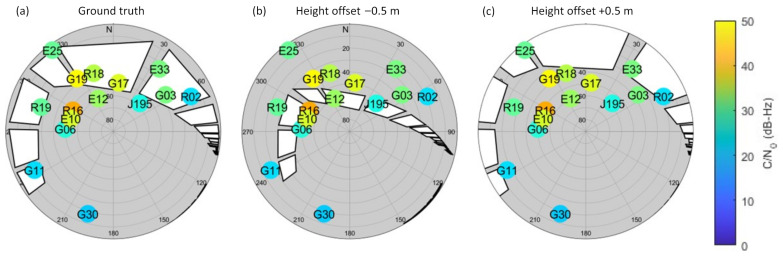
Difference of skymasks: (**a**) The skymask generated according to ground truth. (**b**) The skymask with height offset of −0.5 m. (**c**) The skymask with height offset of +0.5 m.

The process begins by distributing numerous position hypothesis candidates throughout the 3D space defined by an indoor floor plan. This 3D sampling is critical since satellite visibility features, for example, the elevation angle, can change rapidly and are sensitive to height variations, even for the same 2D position. From Figure 5, the skymasks generated with different height offsets result in different window patterns and various satellite visibility classifications. Therefore, the candidates are propagated in 3D space instead of in the 2D domain.

For each 3D candidate e,n,u with distinct coordinates in the ENU direction, satellite visibility is predicted based on the corresponding generated skymask. The probability of signal loss during propagation is evaluated using the $C/N_{0}$ level to mitigate the NLOS reception effect. A visibility similarity score $s_{e,n,u}$ is calculated using (2), in accordance with the 3DMA positioning algorithm in [14]. This score quantifies the match between the predicted LOS visibility from the building geometry $P_{LOS,BB}$ and the observed GNSS signal strength $P_{LOS,C/N_{0}}$. Since the framework focuses mainly on 2D positioning, the scores of candidates at different heights but the same 2D location are averaged to produce a single score for that 2D position using (3).

(2) $$s_{e,n,u}=P_{LOS,C/N_{0}}P_{LOS,BB}-\left(1-P_{LOS,C/N_{0}}\right)\left(1-P_{LOS,BB}\right)$$

where

$$P_{LOS,C/N_{0}}=\left\{\begin{array}{cc} P_{min} & \mathrm{if}\;C/N_{0}\leq {C/N_{0}}_{min}\\ a_{2}C/N_{0}+a_{1}C/N_{0}+a_{0} & \mathrm{if}\;{C/N_{0}}_{min}<C/N_{0}<{C/N_{0}}_{max}\\ P_{max} & \mathrm{if}\;C/N_{0}\geq {C/N_{0}}_{max} \end{array}\right.$$

and

$$P_{LOS,BB}=\left\{\begin{array}{cc} P_{max} & \mathrm{if}\;LOS\\ P_{min} & \mathrm{if}\;NLOS \end{array}\right.$$

(3) $$s_{e,n}=\frac{1}{N_{u}}\sum_{u}s_{e,n,u}$$

where $a_{2}$, $a_{1}$, and $a_{0}$ are coefficients and $N_{u}$ is the number of position hypothesis candidates at each pair of 2D position in the height domain. In the experiment, 3D candidates are points of the self-scanned point cloud sampled every 0.1 m in the horizontal direction and every 0.1 m in the vertical direction. $N_{u}$ includes only specific candidates to reduce the processing load. The candidates are located within the sampling radius (10 m or 15 m) within the current location, limiting the location of the candidates to be within the corridor or, otherwise, the candidates within double of the norm of the selected cluster’s uncertainty value are counted as valid candidates. The values of the variables in this study follow the one suggested in [14]. Table 1 concludes the parameters.

It is noted that at the first epoch k=0, the candidate position lacks the height coordinate *u*. It is assumed that the user height is at 1m above the ground level initially, simulating that the device is held by the user or is in the user’s pocket. For subsequent epochs, the height coordinate is measured by the IMU in the PDR system so no assumption about the user’s vertical motion is made.

**Figure 6:**
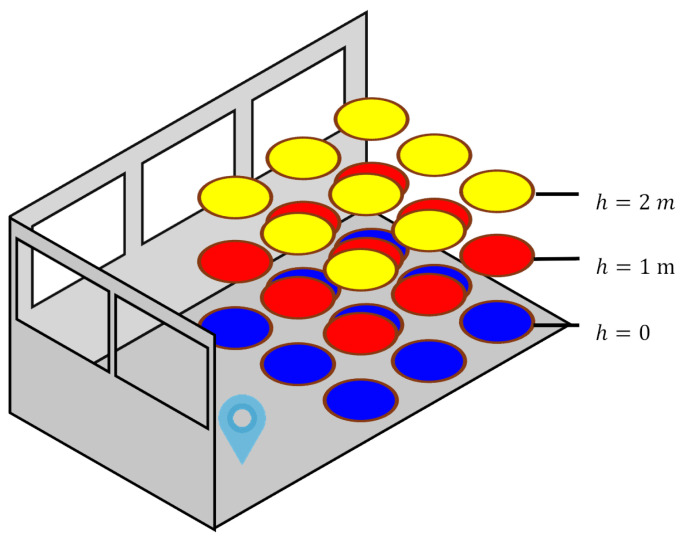
Positioning hypothesis candidates sampling in 3D. Position hypothesis candidates (circular disks) are distributed from the previous position (map pin) in the 3D space. They are coloured by their similarity scores.

Figure 6 simulates the propagation of candidates over the 3D space. In the figure, each disk represents a candidate colored by its corresponding similarity score calculated by Equation (3).

### 3.2. Position Hypothesis Candidate Clustering

Once scores are assigned, candidates are filtered to retain only those with scores above a threshold, representing the most probable locations. In this study, candidates belong to the upper 20% score quantile are preserved for optimal performance. These high-scoring candidates are then segmented into distinct clusters based on the Euclidean distance between them. This clustering step helps group plausible but geographically separate position hypotheses. For each resulting cluster, a weighted average position $x_{k,c}$ and its corresponding variance–covariance matrix $\sum_{k,c}$ are calculated by (4) and (5), respectively. At the initial epoch k=0, the position with the highest average score is selected as the starting position.

(4) $$x_{k,c}=\frac{\sum P_{k,j\in c}\cdot s_{k,j\in c}}{\sum s_{k,j\in c}}$$

(5) $$\sum_{k,c}=\frac{1}{n}{\left(p_{k,c}-x_{k,c}\right)}^{T}\left(p_{k,c}-x_{k,c}\right)$$

where *k* is an epoch, *j* is a candidate, and *c* is a cluster.

### 3.3. PDR Estimation

In parallel with the SM process, stride length and heading derived from PDR are used to estimate the user’s displacement based on inertial data. The formulae in [28] are adopted for these purposes.

Accelerometer readings are first passed through a low-pass filter (LPF) to calculate ${\hat{a}}_{k}$. Steps are detected by identifying dips in the vertical acceleration that fall below a predefined threshold, i.e., Equation (7). After that, the stride length *l* is estimated using an equation based on the maximum and minimum vertical acceleration during a stride (Equation (8)). The heading ψ is derived after applying Principal Component Analysis (PCA) to the accelerometer data and calculating the direction with the larger eigenvalue $\lambda^{*}$, which corresponds to the direction of movement using Equation (9). Ultimately, the estimated stride length and heading are combined to calculate the displacement vector $\Lambda x_{PDR,k}$ for each step (Equation (10)). This PDR-derived trajectory serves to propagate the initial location and deliver a temporal constraint for the overall system.

(6) $${\hat{a}}_{k}=\left(1-\alpha^{acc}\right)a_{k}+\alpha^{acc}{\hat{a}}_{k-1}$$

where $\alpha^{acc}$ is a weighting parameter between 0 and 1, $a_{k}$ is the acceleration of the current epoch, and ${\hat{a}}_{k-1}$ is the filtered acceleration of the previous epoch. In this study, $\alpha^{acc}=0.84$.

(7) $${\hat{a}}_{v,k}\leq {threshold}_{dip}$$

(8) $$l=K_{w}{\left(a_{v,max}-a_{v,min}\right)}^{\frac{1}{4}}$$

where $K_{w}$ is a factor for unit conversion, and $a_{v,max}$ and $a_{v,min}$ are the maximum and minimum vertical acceleration measured during the stride, respectively. In this study, it is assumed that $K_{w}=0.6531$ for all users.

(9) $$\psi =\tan(\frac{v_{\lambda^{*},e}}{v_{\lambda^{*},n}})$$

(10) $$\Lambda x_{PDR,k}=l\cdot \left[\sin\psi \cdot \cos\psi \right]$$

### 3.4. Cluster Selection with EKF Integration

The final stage integrates the I-SM clusters with the PDR displacement estimate using an EKF. The state and covariance from the previous epoch are propagated forward using the PDR displacement to generate the predicted state $x_{k}^{\prime }$ and covariance $P_{k}^{\prime }$ by (11) and (12), respectively. The algorithm then compares this PDR-predicted position with the positions of all candidate clusters generated by I-SM. The cluster $c^{*}$ that yields the minimum Mahalanobis distance to the predicted state is selected as the most probable measurement for the current epoch. This is a significant step as the temporal constraint from PDR helps resolve ambiguity and select the correct position cluster with Equation (13), particularly when navigating turns or experiencing GNSS outages. The EKF calculates the Kalman gain $K_{k,c^{*}}$ using the covariance of the predicted state $P_{k}^{\prime }$ and the selected cluster $\sum_{k,c^{*}}$ (Equation (14)). The gain is a weighting factor that determines how much the system should trust the new measurement versus its prediction. Ultimately, the state $x_{k,c^{*}}$ and covariance $P_{k}$ are updated with information from the selected cluster to produce the final and refined position estimate, which is less uncertain, using Equation (15) and Equation (16), respectively.

(11) $$x_{k}^{\prime }=Ax_{k-1}+B\Lambda x_{PDR,k}$$

(12) $$P_{k}^{\prime }=AP_{k-1}A^{T}+Q_{k}$$

(13) $$c^{*}=\underset{c}{\arg\min}\left(x_{k-1}-x_{k,c}\right)\sum_{c}^{-1}{\left(x_{k-1}-x_{k,c}\right)}^{T}$$

(14) $$K_{k,c^{*}}=P_{k}^{\prime }H^{T}{(HP_{k}^{\prime }H}^{T}+\sum_{k,c^{*}}{)}^{-1}$$

(15) $$x_{k,c^{*}}=x_{k}^{\prime }+K_{k,c^{*}}\left(x_{k,c^{*}}-Hx_{k}^{\prime }\right)$$

(16) $$P_{k}=\left(I-K_{k,c^{*}}H\right)P_{k}^{\prime }$$

In this experiment, clusters are treated independently at each epoch, and the cluster selection does not evolve over consecutive epochs. If the PDR-based solution lies far from the 3DMA candidate cluster, the point with the smallest covariance is chosen as the final estimate. In the case where no valid clusters are identified by I-SM, the EKF solely relies on the PDR estimate to yield the final state and its covariance.

In Equation (11), the state $x_{k}^{\prime }$ predicts the 2D position, i.e., east and north coordinates, of the point in the next epoch. This uses the previous point $x_{k-1}$ and PDR displacement estimate $\Lambda x_{PDR,k}$. *A* is a 2×2 identity matrix, i.e., $\left[\begin{array}{cc} 1 & 0\\ 0 & 1 \end{array}\right]$. *B* is a 2×2 diagonal matrix of Δt, i.e., $\left[\begin{array}{cc} \Delta t & 0\\ 0 & \Delta t \end{array}\right]$, where Δt=1s. Equation (12) includes the process noise covariance of PDR $Q_{k}={\left[\begin{array}{cc} 0.5 & 0\\ 0 & 0.6 \end{array}\right]}^{2}$. These empirical values simulate the noise of the IMU in the PDR system. In Equation (14), *H* is a 2×2 identity matrix. The measurement noise covariance matrix $\sum_{k,c^{*}}$ refers to the product of the covariance matrix of the cluster selected at the epoch *k* and constants ${\left[\begin{array}{cc} 24 & 0\\ 0 & 26 \end{array}\right]}^{2}$.

## 4. Results

The performance of the 3DMA GNSS concept, integrating I-SM with PDR, is evaluated, focusing predominantly on 2D positioning accuracy. The results highlight the benefits of the proposed integrated approach while also identifying the critical factors that influence its performance.

The results are compared with the ground truth to present their accuracies. The ground truth is based on the trajectory of the user recorded by the handheld equipment mentioned in Section 2.4.

In this study, three runs are conducted along sections of the corridor with different devices held by various users. Details of these experiments are presented in Table 2.

In each run, three RMSE values are observed, each with the following approaches:

- PDR (first epoch initialized by the ground truth);
- Single epoch I-SM;
- EKF: I-SM+PDR.

**Figure 7:**
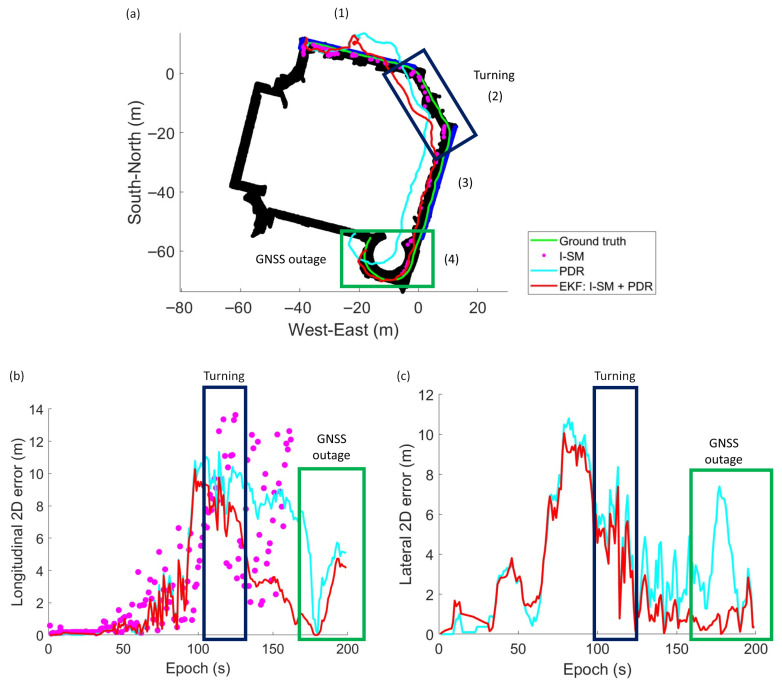
Experiment results of Run 1: (**a**) The ground truth and position solutions derived from the three algorithms. (**b**) Longitudinal 2D error of approaches. (**c**) Lateral 2D error of approaches.

**Figure 8:**
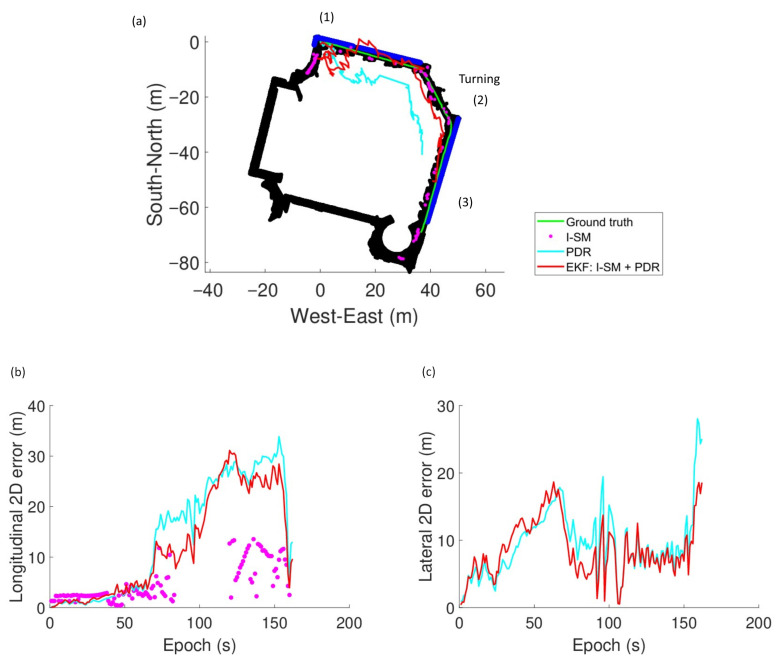
Experiment results of Run 2: (**a**) The ground truth and position solutions derived from the three algorithms. (**b**) Longitudinal 2D error of approaches. (**c**) Lateral 2D error of approaches.

**Figure 9:**
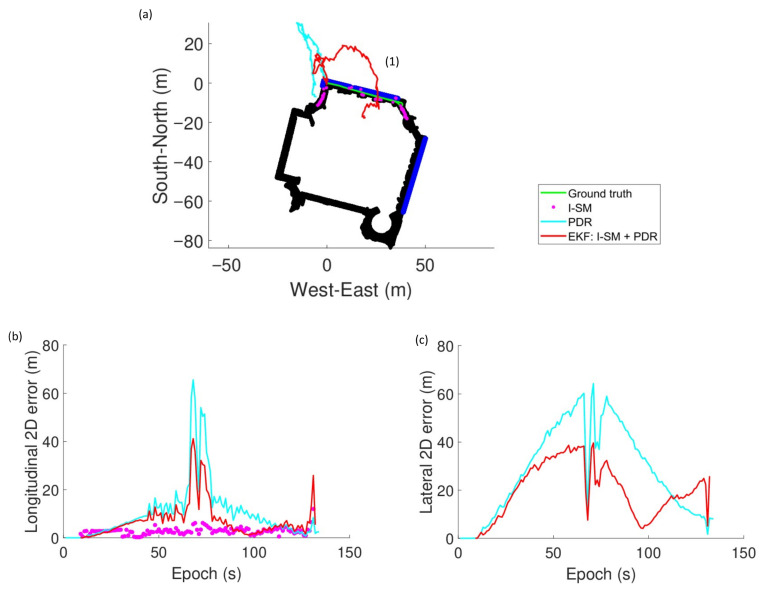
Experiment results of Run 3: (**a**) The ground truth and position solutions derived from the three algorithms. (**b**) Longitudinal 2D error of approaches. (**c**) Lateral 2D error of approaches.

The results of the experiments are summarized in Table 3 and Figure 7, Figure 8 and Figure 9. The black regions and blue lines in Figure 7a, Figure 8a, and Figure 9a indicate the corridor and windows of the building.

Experiments demonstrate the effectiveness of the integrated I-SM and PDR approach. The EKF method often outperforms both standalone PDR and single epoch I-SM across runs, especially in Run 1, achieving potentially lower RMSE values and higher availability. This indicates that the fusion of GNSS visibility information with inertial data significantly enhances positioning accuracy, particularly in challenging indoor environments where GNSS signals are weak or obstructed.

RMSE values vary across runs, reflecting differences in device quality, user movement patterns, and environmental conditions. Run 1 achieves the best performance with an RMSE of 5.52 m using the EKF approach, while Runs 2 and 3 exhibit larger absolute and relative errors. This is likely due to the heading error of the PDR system. As shown in Figure 8a and Figure 9a, the heading of the user is incorrectly estimated at the beginning of the run. It results in significant deviations in the estimated trajectory in both the longitudinal and lateral directions, for example, during the period of epochs 60 to 80 s in Run 2 and 200 s to 300 s in Run 3. This highlights the importance of accurate heading estimation in PDR systems for effective integration with GNSS-based methods. Despite the large error of PDR heading estimation, the EKF approach still manages to improve positioning accuracy compared to standalone PDR.

Step detection in PDR and the determination of final positions using EKF are time-consuming. Table 4 summarizes the processing time for each run, which was executed offline in MATLAB 9.13.0 (R2022b) [26]. The processing time generally increases with the number of epochs due to the increased computational load.

In this study, runs adopt different processing parameters due to the variations in device capabilities and user behaviors. This adjustment accounts for the increased uncertainty in the PDR estimates observed in these runs, likely due to differences in device IMU quality and user movement patterns. The specific parameters used in each run are detailed in Table 5. It is observed that adjusted dip thresholds may be needed for users with different walking patterns. That value would significantly affect the number of steps detected. For EKF, the sampling radius regulates the number of candidates at the current epoch. Meanwhile, a larger $Q_{k}$ can accommodate higher uncertainty in PDR estimates. These two parameters are important for cluster selection in the EKF process and thus the performance of the framework.

## 5. Evaluation

Beyond the aggregated RMSE values, a closer inspection of the results is necessary to understand the factors influencing the positioning accuracy. The subsequent section analyses the components that contribute to the overall performance, identifying key benefits and challenges.

### 5.1. GNSS Signals

**Figure 10:**
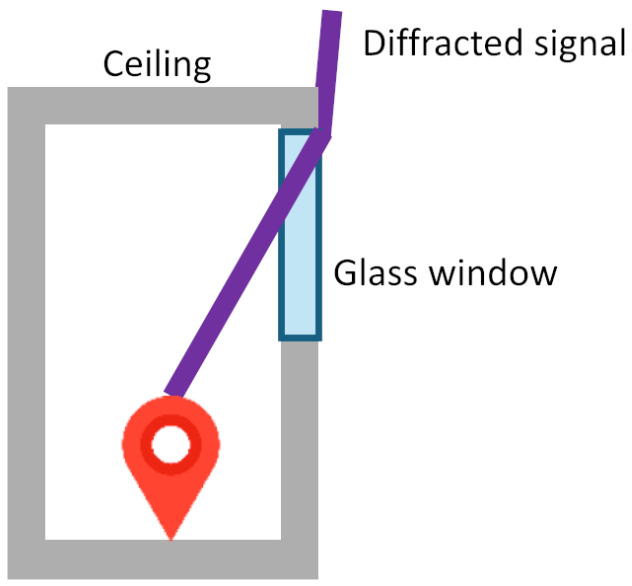
Signal diffraction simulation. Signals may not be direct LOS under the effect.

Firstly, inaccurate $C/N_{0}$ readings often lead to a mismatch between predicted visibility and actual signal conditions. They may be caused by strong diffracted signals, as diffracted and NLOS signals are common in urban environments [2,3]. Those signals usually have $C/N_{0}$ values lower than expected. This complicates satellite visibility predictions and potentially leads to errors in matching. In this study, the received signals are observed to be diffracted when traveling through a window (Figure 10). They cause invalid $C/N_{0}$ values, erroneous scores assigned to position hypothesis candidates, thereby hindering the correct cluster selection.

Then, unexpected non-received signals prompt satellite discrepancies in visibility classification. There were instances where signals were not received even though they were predicted to be present, as well as cases where signals were detected despite being forecasted to be blocked. These discrepancies create confusion in the visibility classification process, undermining the algorithm’s ability to make reliable assessments regarding satellite visibility.

Moreover, positions may not be reliably determined if features (LOS signals) are unevenly distributed. The distinctive visibility features of the LOS signals may sometimes not be evenly distributed as in Figure 3a where most GNSS satellites are located in the northern part. It is more difficult to determine the accurate position, particularly when walking along the corridor where the features might change rapidly.

### 5.2. PDR Integration

The integration of PDR plays a significant role in enhancing the positioning solution. Firstly, PDR supplies temporal constraints on the user’s longitudinal direction along the corridor. From Figure 7b, it is observed that the longitudinal error of the solutions derived by I-SM (purple dots) recorded in (3) during the period of epochs 110 to 150 s is large. Meanwhile, PDR-included solutions (cyan and red lines) are more accurate. Positioning solutions provided by GNSS and I-SM are comparatively inaccurate because GNSS signals can hardly reach the indoor receiver. In addition, the PDR system is capable of predicting a user’s location using previously determined position, the user’s velocity, and heading, without utilizing external GNSS signals. It can predict the distance travelled by the user for each step by exploiting the acceleration data to limit the user’s displacement, all using local instruments. As a result, estimates by or integrated with PDR are closer to the ground truth. In addition, PDR improves cluster selection because it effectively selects the correct cluster of position candidates, especially during challenging scenarios, such as turns and GNSS outages. PDR is able to yield more accurate solutions by providing temporal constraints and propagating initial locations. PDR helps filter possible solutions and maintain continuity in positioning.

However, the effectiveness of PDR is sensitive to its input quality. An incorrect heading estimated by the PDR system significantly degrades the overall positioning performance. In Run 1 (Figure 7), it is noticed that the paths of the two PDR-included methods (cyan and red lines) deviate from the ground truth due to an inappropriate heading estimation. This causes unexpected large single epoch errors in both directions, especially laterally, in the first 100 s. The issue is critical in Runs 2 and 3, as shown in Figure 8 and Figure 9. These underscore the importance of accurate heading determination for robust PDR integration as the PDR-derived heading information would affect the accuracy of the framework in several aspects. Firstly, the estimated solution deviates a lot from the user’s trajectory if the heading is largely erroneous. Secondly, the correct candidate cluster cannot be selected if the heading is inaccurate. These deteriorate positioning performance.

### 5.3. Other Issues

The proposed algorithm requires the receiver’s capability of tracking weak signals. The ability to effectively track and utilize weak signals is paramount in indoor environments where signal strength is often attenuated [29]. Higher capability increases the availability of the proposed framework and the accuracy of the positioning solution.

**Figure 11:**
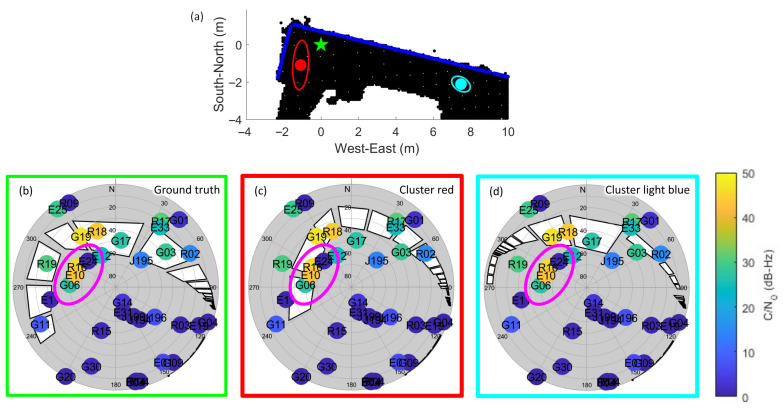
Local minima issue: (**a**) Top view of the ground truth (green), red cluster and light blue cluster. (**b**) The skymask of the ground truth. (**c**) The skymask of the red cluster. (**d**) The skymask of the light blue cluster.

It is noted that the local minima issue occurs when multiple candidate clusters obtain similar satellite visibility scores. Figure 11a shows the locations of two clusters of a hypothesis position, the red cluster which is the closest to the ground truth, and light blue cluster which is farthest. When comparing the two skymasks of corresponding clusters in Figure 11c,d, the four satellites circled in purple represent the key to match the satellite visibility. In the circle, it is believed that the solution should be closer to the window, i.e., the red cluster, due to strong diffracted signals. However, a signal from one of the satellites is not received. Thus, this angular area might be blocked. As all received signals originate from the north and approximately the same elevation angle, the scores obtained by the red and light blue clusters might be very similar. As a result, the inappropriate cluster is selected. This suggests the importance of correct signal reception in the framework.

These observations show that the 3DMA GNSS concept leads to promising indoor positioning, particularly with the aid of 3D models and PDR. It is essential to robustly manage various error sources related to signal reception, model accuracy, and sensor integration to achieve higher accuracy and reliability of indoor positioning.

## 6. Discussion

When using 3DMA GNSS for positioning in indoor environments, understanding the error budget is important as it directly impacts the accuracy and reliability of the framework. The primary features leveraged for matching predictions in this system are visibility classification and signal strength. Therefore, errors in these aspects significantly degrade positioning performance.

The error budget can be categorized according to the components that contribute to the positioning framework.

### 6.1. Errors Related to 3D Models

The accuracy of the 3D model, which is used for hypothesis position candidate sampling in the 3D domain and subsequently for visibility prediction, is paramount.

**Figure 12:**
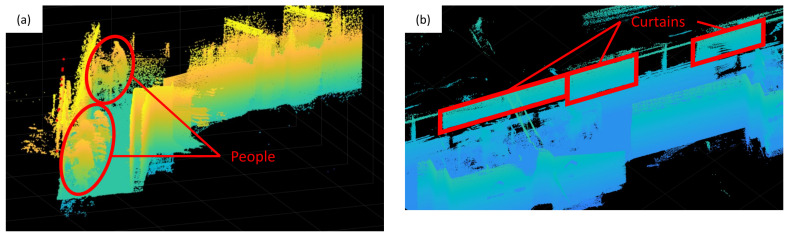
Errors related to 3D models: (**a**) The inclusion of dynamic objects, for instance, humans, in the point cloud. (**b**) Signal-penetrable materials, such as curtains, appear as solid obstructions in the point cloud. Points are coloured by their intensities.

The inclusion of dynamic obstructions, such as humans, leads to inaccuracies in the satellite visibility classification. These temporary elements alter the expected LOS or NLOS properties for satellite signals to a great extent. Figure 12a shows the inclusion of people in the point cloud. In addition to the movable obstacles, signal-penetrable materials, for example, curtains, are also captured. They appear as solid obstacles in a point cloud. However, in reality, signals can penetrate those materials and reach the receiver. Figure 12b illustrates the effect of the scanned curtains in the point cloud. With the above components in point clouds, GNSS signals are believed to be blocked and therefore cause discrepancies between the predicted satellite visibility from the 3D model and the actual signal reception.

In this experiment, the 3D model generated by laser scanning includes dynamic objects and signal-penetrable materials. The mesh is not post-processed to remove these objects. Interior environments of buildings can be retrieved from BIM models created during the construction phase or as-built models. These representations are less likely to contain temporary objects and materials that affect signal propagation.

Furthermore, the parameters for laser scanning affect the accuracy of 3D models. One of the significant parameters is the point density of point clouds. Denser points indicate better capture and representation of the environment. This setting creates denser spatial data, records complex geometries and subtle details of objects, and reduces environmental noise in the scanning. In indoor spaces, high point density should be adopted to capture complicated areas.

These inaccuracies in the 3D model directly affect the satellite visibility classification, which is recognized as a key feature for matching predictions.

### 6.2. Errors Related to Observations

The actual observations, including satellite visibility and received signal strength, are also critical to matching predictions and position determination.

Signal strength sensitivity and resolution are critical factors in the performance of the algorithm. This algorithm requires accurate measurement and resolution of signal strength. Otherwise, it can lead to incorrect comparisons with the predicted characteristics of the signals. Such discrepancies can significantly impact the overall effectiveness of the positioning system, as the algorithm relies on precise signal strength data to make informed decisions.

Similarly, the correctness of satellite visibility plays a crucial role in the accuracy of the positioning framework. There can be unexpected instances where signals are not received, or conversely where signals are received despite being predicted to be blocked. These errors in satellite visibility can introduce further complications in the positioning process, undermining the reliability of the system. Addressing these challenges is essential for improving the overall accuracy and robustness of GNSS performance in various environments.

These observational errors are fundamental to the matching process.

### 6.3. Other Contributing Error Factors from Positioning Results

As mentioned above, incorrect heading, mistaken $C/N_{0}$ values, diffracted GNSS signals, unevenly distributed LOS signals, and the capability of tracking weak signals are factors contributing to the overall error budget.

In addition to those factors, the relative positioning error also impacts the result. The width and height of the corridor in the study area are generally 2 m or 2.5 m, which means that substantial errors in any direction can lead to a dramatically different position longitudinally or laterally. For example, if the lateral error exceeds 2 m, the positioning system may incorrectly indicate that the user is located on the opposite side of the corridor or, in some cases, even outside the corridor or building entirely. Such inaccuracies highlight the critical importance of minimizing relative positioning errors to ensure reliable and accurate location determination in constrained environments.

In summary, achieving reliable indoor positioning with 3DMA GNSS requires a comprehensive consideration of these error sources. The accuracy of 3D models, the fidelity of satellite visibility and signal strength observations, and the proper integration with complementary systems, such as PDR, are indispensable requirements for minimizing positioning errors in indoor settings.

## 7. Conclusions and Future Directions

### 7.1. Conclusions

This study investigates the feasibility of using the 3DMA GNSS concept for indoor positioning, particularly in GNSS-accessible indoor areas. The SM technique is introduced into indoor positioning using satellite visibility classification and signal strength during matching predictions. PDR is integrated to provide an optimized solution. This study demonstrates that the integration of I-SM and PDR effectively improves positioning accuracy in indoor areas.

Key limitations of this integrated approach include the availability of accurate 3D models and quality of GNSS observations. Inaccurate 3D models may lead to incorrect satellite visibility prediction and cluster selection. Performance is compromised if the quality of GNSS measurements is poor. An incorrect heading from PDR and the receiver’s capability to receive and resolve attenuated GNSS signals would also negatively affect the results.

### 7.2. Future Directions

To enhance the accuracy of positioning solutions in indoor environments, future work should focus on super-long coherent tracking, which could improve the ability to track weak signals that are prevalent indoors, as explored in [30], and investigate the efficiency and robustness of the 3D sampling process. Meeting the 2 to 2.5 m positioning accuracy requirements in indoor environments and determining the height is also significant.

Additionally, broad implementation, for instance, LiDAR-based 3D SLAM in [31], in automated 3D model generation and maintenance should be studied. This improves the practicality and scalability of the 3DMA GNSS concept.

## Figures and Tables

**Table 1:** Tuning parameters in [14] are adopted in this experiment.

	${C/N_{0}}_{\min}$ (dB-Hz)	${C/N_{0}}_{\max}$ (dB-Hz)	$P_{\min}$	$a_{0}$	$a_{1}$ (dB-Hz^−1^)	$a_{2}$ (dB-Hz^−2^)	$P_{\max}$
Value	27	44	0.15	0.4549	−0.0444	0.0012	0.85

**Table 2:** Experiment details.

Run	Device	Route
1	Xiaomi 8	(1) > (2) > (3) > (4)
2	Google Pixel Pro 9 XL	(1) > (2) > (3)
3	Google Pixel Pro 9 XL	(3) > (2) > (1)

**Table 3:** Experiment results (2D RMSEs).

Run	PDR	Single Epoch I-SM	EKF: I-SM + PDR
1	7.44	7.79 (availability: 81.4%)	5.52 (availability: 100.0%)
2	21.16	10.20 (availability: 76.5%)	18.77 (availability: 100.0%)
3	37.16	10.41 (availability: 91.8%)	25.37 (availability: 92.5%)

**Table 4:** Experiment statistics.

Run	Time Taken for PDR (s)	Time Taken for EKF (s)	Number of Epochs
1	401	2081	162
2	412	1870	160
3	405	1375	123

**Table 5:** Experiment parameters.

Run	PDR Dip Threshold (m^2^/s^2^)	Sampling Radius (m)	$Q_{k}$
1	7.5	10	${\left[\begin{array}{cc} 0.5 & 0\\ 0 & 0.6 \end{array}\right]}^{2}$
2	10	15	${\left[\begin{array}{cc} 0.5 & 0\\ 0 & 0.6 \end{array}\right]}^{2}$
3	10	15	${\left[\begin{array}{cc} 0.5 & 0\\ 0 & 0.6 \end{array}\right]}^{2}$

## Data Availability

The Spatial Data 3D-BIT00 presented in the study are openly available on Common Spatial Data Infrastructure at https://portal.csdi.gov.hk/geoportal/?lang=undefined&datasetId=landsd_rcd_1637306559892_42396, accessed on 12 November 2025.

## Abbreviations

The following abbreviations are used in this manuscript:

GNSS	Global Navigation Satellite System
3DMA	Three-Dimensional Mapping-Aided
PDR	Pedestrian Dead Reckoning
I-SM	Indoor Shadow-matching
EKF	Extended Kalman Filter
RMSE	Root mean square error
UWB	Ultra-Wideband
BLE	Bluetooth low energy
AP	Access points
SM	Shadow-matching
LBR	Likelihood-based ranging
$C/N_{0}$	Carrier-to-noise ratio
LOS	Line-of-sight
NLOS	Non-line-of-sight
UCL	University College London
BLV	People with blindness and low vision
VIS^4^ION	Visually Impaired Smart Service System for Spatial Intelligence and Navigation
IMU	Inertial measurement unit
FGO	Factor graph optimization
BIM	Building Information Modeling
LiDAR	Light Detection and Ranging
PolyU	The Hong Kong Polytechnic University
SLAM	Simultaneous Localization and Mapping
LPF	Low-pass filter
PCA	Principal Component Analysis
